# Agro-Morphological, Yield, and Genotyping-by-Sequencing Data of Selected Wheat (*Triticum aestivum*) Germplasm From Pakistan

**DOI:** 10.3389/fgene.2021.617772

**Published:** 2021-04-13

**Authors:** Madiha Islam, Abdullah, Bibi Zubaida, Nageena Amin, Rashid Iqbal Khan, Noshin Shafqat, Rabia Masood, Shahid Waseem, Jibran Tahir, Ibrar Ahmed, Muhammad Naeem, Habib Ahmad

**Affiliations:** ^1^Department of Biotechnology and Genetic Engineering, Hazara University, Mansehra, Pakistan; ^2^Department of Biochemistry, Quaid-i-Azam University, Islamabad, Pakistan; ^3^Institute of Molecular Biology and Biotechnology, Bahauddin Zakariya University, Multan, Pakistan; ^4^Institute of Horticultural Sciences, University of Agriculture, Faisalabad, Pakistan; ^5^Department of Agriculture, Hazara University, Mansehra, Pakistan; ^6^Department of Botany, Hazara University, Mansehra, Pakistan; ^7^Alpha Genomics Private Limited, Islamabad, Pakistan; ^8^Terrestrial Bioscience New Zealand Limited, Auckland, New Zealand; ^9^Federal Seed Certification and Registration Department, Islamabad, Pakistan

**Keywords:** wheat, insertions and deletions, substitutions, genetic diversity, genotyping-by sequencing

## Introduction

Wheat (*Triticum aestivum* L.) is the staple food crop for about 30% of the world's population and contributes over 20% of calories from food (Shewry and Hey, 2015). Current global wheat yield should be doubled to feed a projected human population of 9 Billion by 2050 (Ray et al., 2013). Major challenges that hamper the target of significantly increasing yield include climatic changes, reduction in arable land availability, changes in socio-economic conditions of people in developing countries, loss of biodiversity, and biotic and abiotic stresses (Godfray et al., 2010). The target of yield increase can be achieved by investigating and utilizing the genetic diversity in available wheat germplasm, improving cultivar genetics and crop management practices (Godfray et al., 2010; Philipp et al., 2019).

Genetic diversity provides a foundation for crop improvement (Govindaraj et al., 2015) in order to develop varieties that have a better yield as well as resistance to biotic and abiotic stresses (Khan et al., 2015). Assessment of genetic diversity also helps to understand genomic composition, identify genes for vital traits, conserve and classify genetic variation in plant germplasm, and develop techniques for plant propagation (Khan et al., 2015). Since frequent use of few parents or less diverse genotypes leads to genetic erosion by producing progenies with low heterozygosity and/or inbreeding depression, it is critical to determine genetic diversity in the intended parental lines before starting a breeding program (Tar'an et al., 2005). The progenies of parents with low genetic diversity may quickly become prone to biotic and abiotic stresses (Govindaraj et al., 2015; Joukhadar et al., 2017). Conversely, using diverse parental lines or genotypes can produce progenies of desirable genetic makeup that have the tolerance to biotic and abiotic stresses, and that produce higher grain yields (Tar'an et al., 2005).

Agronomic and morphological data have been widely used to screen wheat varieties that are tolerant to stress, including drought (Ali et al., 2013), rust (Singh et al., 2005; Afzal et al., 2008; Luo et al., 2009; Chen et al., 2014), salinity (Zafar et al., 2015), and spot blotch (Jamil et al., 2018). Molecular markers were extensively used to evaluate the genetic diversity and population structure of wheat germplasm (Du et al., 2002; Khan et al., 2005; Ahmed et al., 2010; Akhunov et al., 2010; Sobia et al., 2010; van Poecke et al., 2013; Manickavelu et al., 2014; Zeshan et al., 2016). Studies using randomly amplified polymorphic DNA (RAPD) markers demonstrate narrow genetic backgrounds in most varieties introduced by the same research institutes (Mukhtar et al., 2002; Ahmed et al., 2010). RAPD markers, however, can be problematic in terms of reproducibility and reliability, which can lead to inconsistent and/or weakly supported inferences (Penner et al., 1993). Single nucleotide polymorphisms (SNPs) are the most abundant polymorphism that exist in plant genomes (Batley and Edwards, 2007). SNPs are appropriate for investigating marker-trait association, analyzing genetic polymorphism, mapping quantitative trait loci (QTLs), studying population structure, and genomic selection. However, many SNPs are required to cover a significant part of the genome (Kumar et al., 2012). Recent advancements in high-throughput sequencing coupled with the introduction of the genotyping-by-sequencing (GBS) technique has made it possible to identify genome-wide SNPs in a cost effective manner. These SNPs are useful in crop breeding, DNA fingerprinting, tagging of resistance genes for biotic and abiotic factors, and analyzing genetic diversity (Elshire et al., 2011; Edae et al., 2014; He et al., 2014; Perea et al., 2016; Jamil et al., 2018, 2019). For genomic DNA digestion, the restriction endonucleases utilized in GBS reduce genomic complexity, thereby enabling easier analyses of large and complex genomes such as wheat. Wheat is an allohexaploid with 42 chromosomes and has a genome size up to 17 GB (Clavijo et al., 2017). Breeders can benefit from these cost-effective informative markers during the selection of desirable wheat offspring (Alipour et al., 2017).

**Figure 1:**
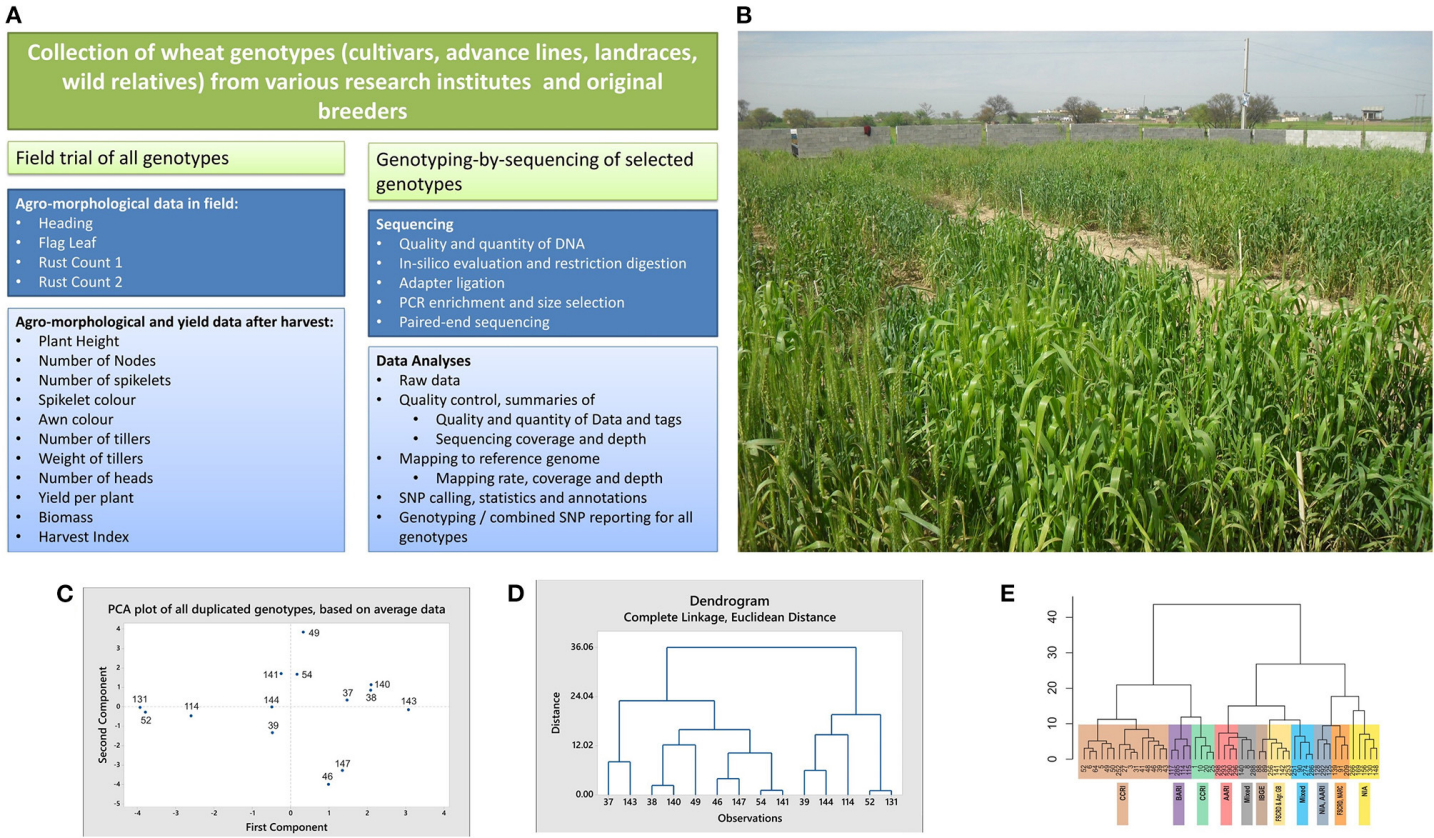
Schematic workflow, field snapshot, multivariate analyses, and dendograms of wheat cultivars. **(A)** Schematic workflow of the study. **(B)** A snapshot of the field. Different genotypes visible in the field were cultivated in blocks for recording agro-morphological and yield data. **(C,D)** Results of the multivariate analyses, showing clustering of the duplicated genotypes. Average data of all plants per genotype was used for these analyses. The duplicate genotypes (IDs in brackets) include Sahar (37 and 143), Faisalabad 2008 (38 and 140), Lasani 2008 (39 and 144), Marvi 2000 (46 and 147), Chakwal 50 (49 and 114), Galaxy (54 and 141), and TD-1 (52 and 131). Except for the genotype Chakwal-50, both the PCA plot **(C)** and dendrogram **(D)** tend to cluster together the duplicates in each genotype. **(E)** Dendrogram based on Ward distances, grouping the genotypes into clusters and sub-clusters. Genotypes collected from individual research institutes tend to cluster together. Detailed methodology is provided in Supplementary Material.

Among top wheat-producing countries, Pakistan ranks 4th in Asia and 11th in the world (Saeed et al., 2012). To the best of our knowledge, genetic diversity in Pakistani wheat cultivars, advance lines, and landraces has not been evaluated using GBS markers. Here we report agro-morphological and yield data, along with GBS data in wheat germplasm from Pakistan. A schematic workflow of the overall study is given in Figure 1A. This data will be useful for inferring genetic diversity, population genetics, marker-assisted selection in breeding, genome-wide association studies (GWAS), mapping of rust and drought-resistant genes and other desirable quantitative trait loci (QTL) as well as for planning effective crop breeding programs in the future.

## Methods

### Collection of Genotypes and Field Trial

A total of 104 wheat cultivars (CVs), landraces (LRs), and advance lines (ALs) were collected from different research institutes, breeders, and original collectors of landraces in Pakistan. An additional seven cultivars were collected from separate research institutes to be included as duplicate controls in agro-morphological data. A wild relative, *Triticum monococcum* (genotype ID: 209), was obtained from the Wide Hybridization Department, National Agriculture Research Center, Islamabad, and included in this study. Supplementary Table 1 gives a list of all 112 genotypes (including seven duplicates) for which agro-morphological and yield data were recorded. This table also gives the NCBI sample accession numbers of a subset of 52 genotypes, which were used to generate GBS data. Among the 112 genotypes mentioned in this table, 55 cultivars were also reported in an online Wheat Atlas (http://wheatatlas.org/country/varieties/PAK/0?AspxAutoDetectCookieSupport=1; Accessed on 1st August 2019). Supplementary Table 2 provides the detailed information about the year of release, pedigree and selection details for these cultivars, presence of the semi-dwarf (Rht) gene, and the area for which the cultivar was developed for these 55 common cultivars, as provided in the online Wheat Atlas. The field trial was conducted in a plain field in Mandra, a town located 45 km south of Islamabad, in the Potohar region (arid zone). The geographical coordinates for the site are 33°38′N, 73°26′E. Before sowing, the field was plowed, fertilizer was homogeneously mixed in the soil, and the soil was leveled. Seeds of the genotypes were sown from 15th November 2015 to 20th November 2015. Each genotype was sown in one square meter block, comprising 25 plants (5 rows × 5 columns) except for four genotypes for which <25 seeds per genotype were available (identified in Supplementary Table 1). The sixth row for all blocks comprised a rust spreader cultivar, called Morocco. The genotypes were sown in triplicate, in randomized blocks. Figure 1B gives a snapshot of the field trial.

### Agro-Morphological and Yield Data

Data were recorded in the field as well as after harvest. The field data consists of four qualitative variables. This data was based on the observation and scoring of data of entire blocks; individual plants were given the same score as that of the block for these four variables. At maturity, five plants per block were uprooted from the soil and labeled individually. The plant labeling after the harvest followed the EnRnPn scheme, where “E” showed “Entry” number (1–300 unique genotype IDs among 112 genotypes, as given in Supplementary Table 1), “R” represented replicate number (1–3), and “P” indicated plant number (1–5). For example, E1R2P5 represents entry (genotype ID) number 1, replicate number 2, and plant number 5. This labeling scheme ensured keeping identity of the plants while recording the subsequent qualitative and quantitative data. With few exceptions mentioned below, agro-morphological and yield data were recorded for 15 individual plants (five plants per replicate, in triplicates) per genotype.

### Data Recorded in the Field

The traits or agro-morphological variables for which qualitative data were recorded in the field included heading (H), flag leaves (FL), rust count 1 (RC1), and rust count 2 (RC2). Heading data were recorded at the booting stage for most of the plants in the field, and all data were recorded in a single field visit. The data were scored as 1–8, based on the presence or absence of heads on most of the plants in the entire block. Flag leaf status was recorded as drooping to erect for the entire block and given scores as 1–4. Stripe rust was scored on a scale from 0–9, as reported elsewhere (Dinglasan et al., 2016). Stripe rust was scored twice; first count (RC1) was recorded 29th March 2016 and the second count (RC2) was recorded on 15th April 2016.

#### Data Recorded After Harvesting Plants at Maturity

After maturation, harvesting of the plants started on 30th April 2016 and continued till 15th May 2016. Most of the genotypes (CVs, ALs, and some LRs) were ready to harvest by the end of April; many LRs and some CVs were late in maturity and were harvested in the first and second week of May. Cold adapted LRs from the temperate region of Gilgit in northern Pakistan were the last to reach maturity. Fewer than five plants per block could be collected at maturity for these genotypes (sample IDs: 253, 255, and 256), leading to missing post-harvest data for the rest of the plants. Remaining plants for these genotypes did not reach maturity till the end of May 2016 (one month after the start of harvest) and were abandoned in the field. The following qualitative and quantitative data were recorded after the harvest:

Qualitative data were recorded for Spikelet color (SC) and Awn color (AC). The colors were scored either 1 (red to brown) or 2 (white to amber), as reported by elsewhere (Ormoli et al., 2015).

Quantitative data were recorded for nine variables, including Plant height (PH), Number of nodes (NN), Number of spikelets (NS), Number of tillers (NT), Weight of tillers (WT), Number of heads (NH), Yield per plant (YP), Biomass (B), and Harvest Index (HI). A brief description of each of the quantitative data recorded is given in Supplementary Material.

### Genotyping by Sequencing

Based on economic importance, a sub-set of 52 genotypes (Supplementary Table 3) was selected to generate genotyping-by-sequencing (GBS) data. The varietal evenness for these 52 genotypes was based on the agro-morphological and yield data. Seeds were grown at room temperature in plastic trays (12 inches width × 24 inches length × 2.5 inches depth; 4 × 8 cells) using autoclaved soil and sand mixed 2:1. After 14 days of sowing, leaf tissues from 10 seedlings per sample were harvested and pooled for DNA extraction using the GeneJET Plant Genomic DNA kit (Catalog No. K0791, ThermoFisher Scientific USA). The quality and quantity of DNA were confirmed with 1% agarose gel electrophoresis and uDrop Plate of Multiskan GO (ThermoScientific, USA). DNA samples were lyophilized and shipped to Novogene Inc. Hong Kong for sequencing.

At Novogene, the purity and integrity of DNA were determined with agarose gel, and Qubit® 2.0 fluorometer was used for accurate quantification of DNA concentration. For library construction, all samples contained at least 1.5 ug DNA. MseI and NlaIII restriction endonucleases were selected after *in silico* evaluation to generate >400,000 tags per sample and were employed for digestion of DNA (0.3–0.6 ug). Adapters were ligated to DNA along with a unique barcode for each wheat genotype. All libraries were pooled and subjected to a polymerase chain reaction (PCR) for the enrichment of sequence data. The qualified libraries were sequenced using Illumina high-throughput sequencing with 144 bp paired-end run. Average insert size of 303 bp was determined for each genotype, using Bioanalyzer.

The sequencing data was generated on a HiSeq 2500 instrument. Adapters were trimmed from the ends. Those reads which were either contaminated with library adapters, 10% unknown bases (N) or 50% low-quality bases were not used in downstream analysis. The quality of short reads was assessed using FastQC version 0.11.6 (Andrews et al., 2020) using default parameters. *Triticum aestivum* TGACv1 (Clavijo et al., 2017) was used as a reference genome for mapping the short reads using Burrows-Wheeler Alignment (BWA) version 0.7.1 (Li and Durbin, 2009) with default parameters. The reference genome was downloaded from Ensembl (ftp://ftp.ensemblgenomes.org/pub/release33/plants/fasta/triticum_aestivum/dna; File: Triticum_aestivum.TGACv1.dna.toplevel.fa.gz; date accessed 22nd March 2018).

All variants were filtered using SAMtools version 1.6 (Li et al., 2009) using parameters “–q = 1, –C = 50, –m = 2, –F = 0.002, –d = 1,000.” PICARD version 2.18.0 (Broad Institute, 2018) was used to remove duplicates. To further reduce the error rate in substitutions calling, only those SNPs were selected that had coverage depth higher than 4x and mapping quality higher than 20. ANNOVAR (Wang et al., 2010) was used for the functional annotation of each substitution.

### Data Records

The agro-morphological and yield data are presented in Supplementary Table 3. The table also provides information about the qualitative and quantitative data for 15 plants per genotype (five plants per plot, triplicates), along with the keys used for the qualitative data. Supplementary Figure 1 is a Box-plot representation of the dispersion in the data for all 15 variables studied. Minitab version 18 was used to generate this figure.

All GBS sequencing data and associated BAM files have been submitted in Sequence Read Archive (SRA) of the NCBI repository (NCBI BioProject, 2019) and assigned SRA project number SRP179096. Individual Fastq files were given accession numbers SRR8441393 through SRR8441444; BAM files were given accession numbers SRR8467619 through SRR8467670. In total, 89.036 GB of clean data were produced; per sample data ranged from 1.01 to 2.5 GB. The lowest Phred score value for Q30 was 89.41%. The values of GC content in individual samples ranged from 42.14 to 44.17%. Information about individual samples, quantity, and quality of generated data are provided in Supplementary Table 4 along with details of each wheat variety, numbers of bases generated per sample and their respective quality values. Reference genome mapping information is given in Supplementary Table 5. This table provides a summary statistic of the mapping of short reads to the wheat reference genome.

Supplementary Table 6 gives summary statistics about the variants called (SNPs) for individual genotypes. This table also gives functional attributes of the SNPs and gives the number of transition and transversion mutations. The average number of SNPs per genotype was 364,074 ± 54,479. When SNPs for all genotypes were merged, the total number of SNPs reached 2 Million. These combined SNPs, with exact nucleotide positions on the wheat reference genome, are given in the file “Genotyping and SNPs data” (Islam et al., 2020), available on Figshare. This file contains a complete record of SNPs. The data in each column can be read from left to right–#Chromosome: Chromosome position along the small arm and long arm of the chromosome, #Position: The coordinate position of nucleotide base which showed substitution, #Reference: The nucleotide present in the reference genome, #Allele: The type of substitution in the reference genome showing first the allele present in the reference genome and then the allele present in the sample sequence in the current study, #Gene: The name of the gene in which the substitution exists, #Annopos: Type of substitution according to the location, such as intergenic, genic, intronic, UTR, synonyms and non-synonyms. The next column shows the substitution present in each sample in a diploid form such that GG represents the homozygous condition and AG represents the heterozygous condition.

GBS data generated for various crops including wheat has been used to study genetic diversity, population genetics, phylogenetics (Lateef, 2015; Li et al., 2015; Chung et al., 2017; Elbasyoni et al., 2018), association mapping and genome-wide association studies (Bastien et al., 2014; Arruda et al., 2016; Muqaddasi et al., 2017; Yu et al., 2017; Jamil et al., 2018), linkage map and quantitative trait loci (QTL) mapping (Bielenberg et al., 2015; Verma et al., 2015; Balsalobre et al., 2017; Hussain et al., 2017; Scheben et al., 2017), marker-assisted and genomic selection (He et al., 2014; Scheben et al., 2017). Together with agro-morphological and yield data, GBS data generated for wheat genotypes in this study will be extremely useful in future crop breeding programs. The data will be helpful in the breeding of elite wheat cultivars having high yield and resistance to biotic and abiotic stresses to feed the growing human population.

### Technical Validation

Seven cultivars were included as duplicate controls in the current study for technical validation. The dupicates were collected form different research institutes. Identity of the duplicates, their sources of collection, and description about their comparison in agro-morphological and yield data, as well as GBS data is provided Figures 1C–E and Table 1. Details about the technical validation for varietal evenness is provided in Supplementary Material.

**Table 1:** Pearson's correlations among alleles of the Chakwal-50 replicates (IDs 49 and 114) using GBS data.

**Comparison among alleles**	**Pearson's correlation**
Genotype 49, Allele 1, 2	0.997
Genotype 114, Allele 1, 2	0.996
Genotype 49, Allele 1; Genotype 114 Allele 1	0.493

### Availability of the Wheat Genotypes

Sources of the wheat genotypes collection have been listed in Supplementary Table 1. The source institutes are expected to annually refresh and retain the propagating material, which is essential the viability of germplasm over the years. As per the Plant Breeders' Rights Act 2016 in Pakistan, original breeders of the cultivars and advance lines retain the property rights of their breeding material. In line with this Act, the authors are not authorized to share and disseminate the genotypes covered by the Act. The authors welcome queries from other researchers and potential breeders about the availability and sharing of the genotypes which are not protected by the Act. Where applicable, the respective laws of donor and recipient countries will govern the transfer of the propagating/living material to other countries outside Pakistan.

## Code Availability

Except CASAVA, all software tools used are free to use and publicly available.

## Data Availability Statement

The datasets presented in this study can be found in online repositories. The names of the repository/repositories and accession number(s) can be found in the article/Supplementary Material.

## Author Contributions

MI: study design, sample collection, data recording in the field and after the harvest, data analyses, DNA extractions, and manuscript writing. Abdullah: data collection in the field and after the harvest, data analyses including GBS data, DNA extractions, and manuscript writing. BZ: sample collection and data analyses. NA: data collection after the harvest. RK and SW: data collection in the field and after the harvest. NS and RM: sample collection and data collection after the harvest. JT: GBS data analysis. IA: study design, field work, data collection, data analyses including GBS data, manuscript editing, and study co-supervision. MN: study design, sample collection, field work, data collection, data analyses, and study co-supervision. HA: study design and overall project supervision. All authors contributed to the article and approved the submitted version.

## Conflict of Interest

IA and SW were employed by the company Alpha Genomics Private limited. The remaining authors declare that the research was conducted in the absence of any commercial or financial relationships that could be construed as a potential conflict of interest.
