# Supplementary figures and images for: Agro-Morphological, Yield, and Genotyping-by-Sequencing Data of Selected Wheat (Triticum aestivum) Germplasm From Pakistan
Source: Front Genet. 2021 Apr 13;12:617772. doi: 10.3389/fgene.2021.617772 (PMC8216712; doi:10.3389/fgene.2021.617772)

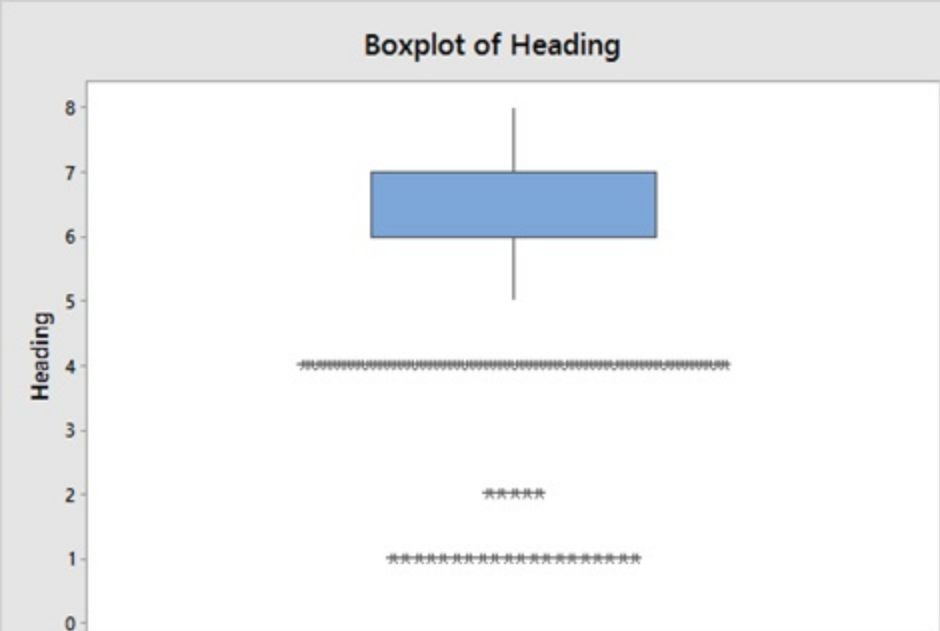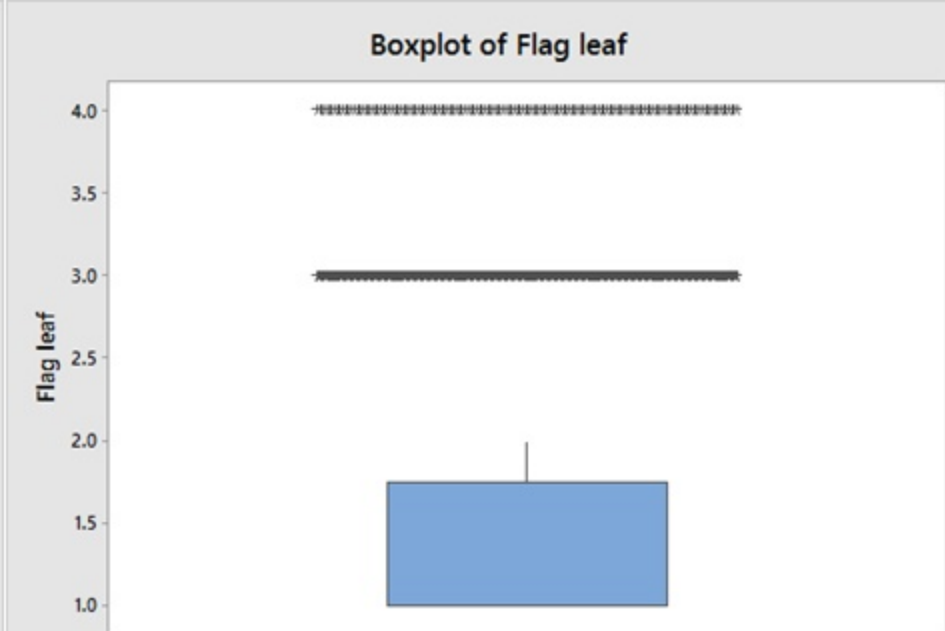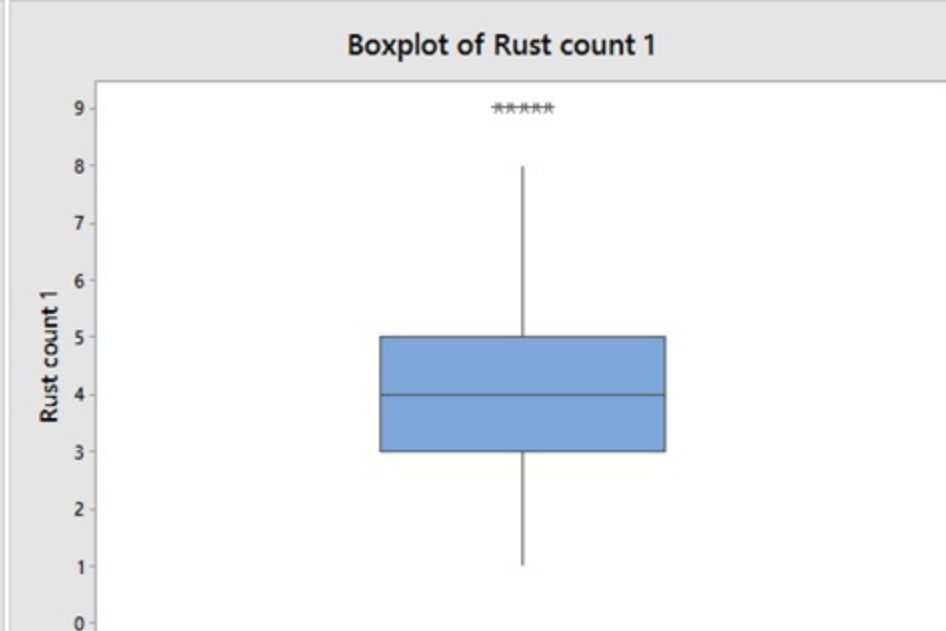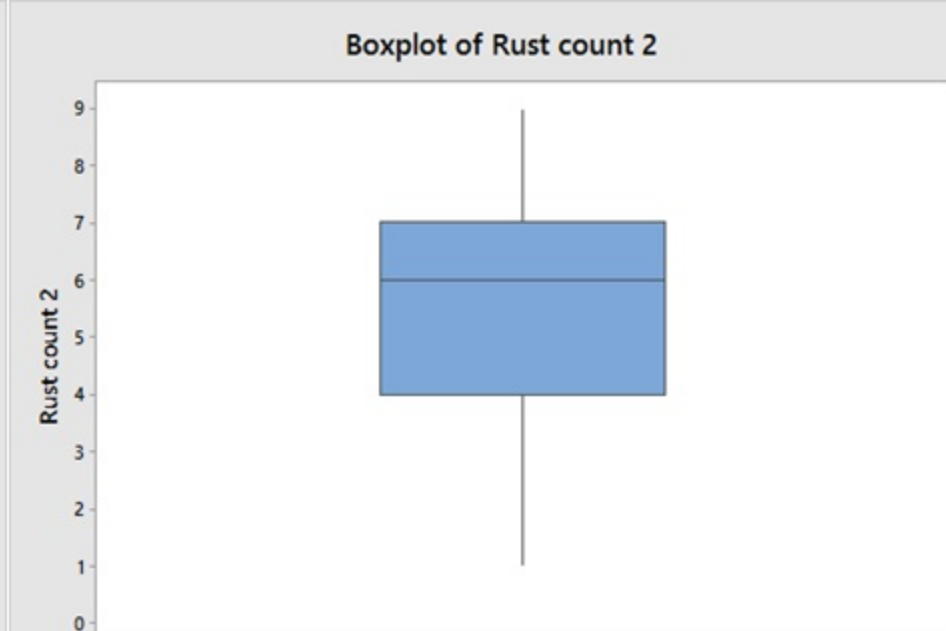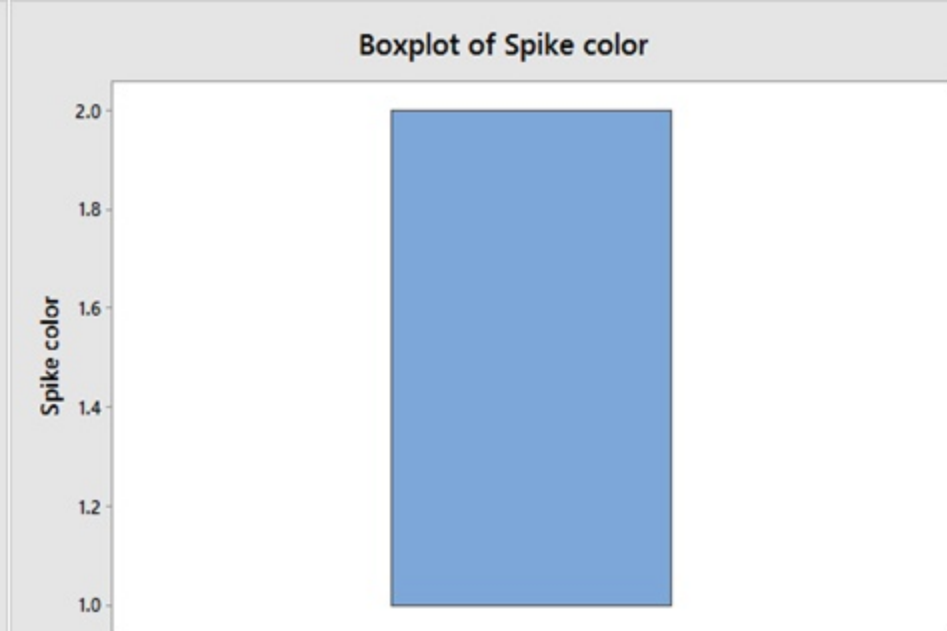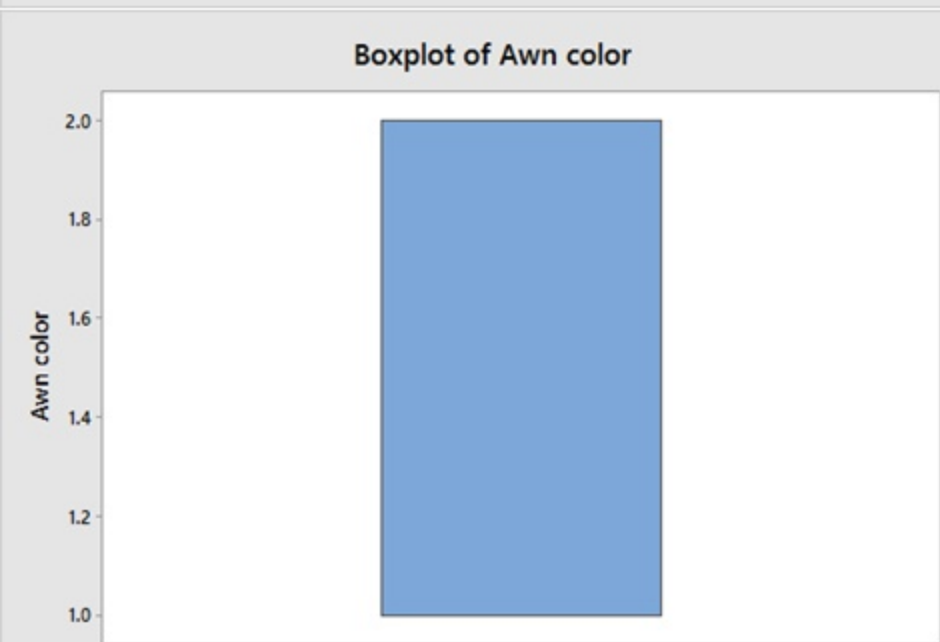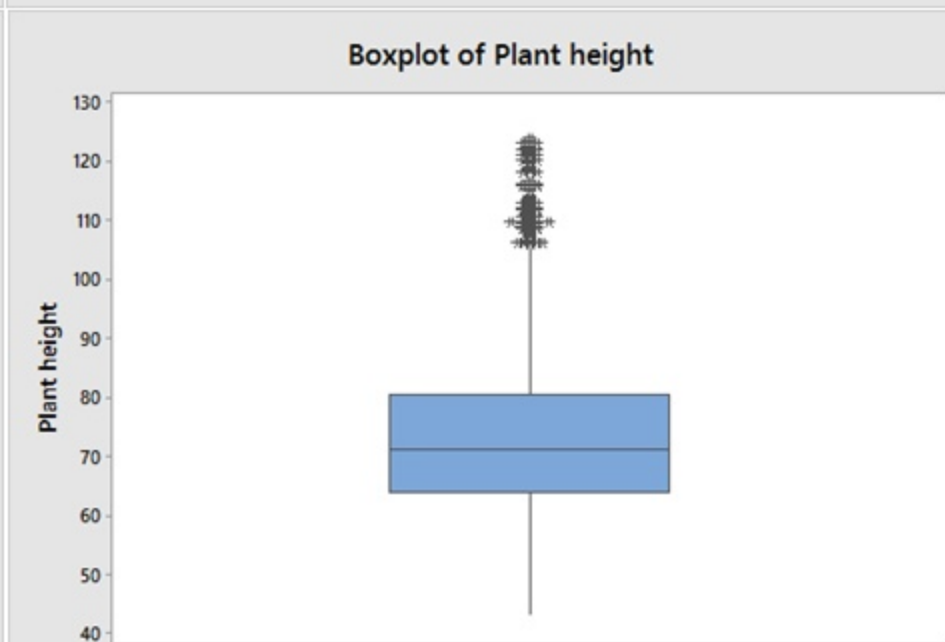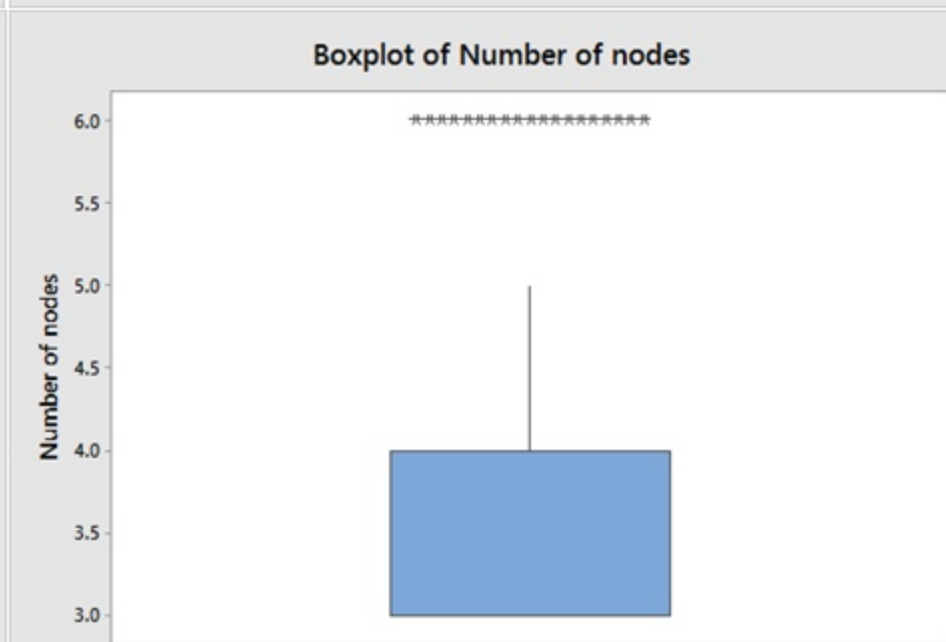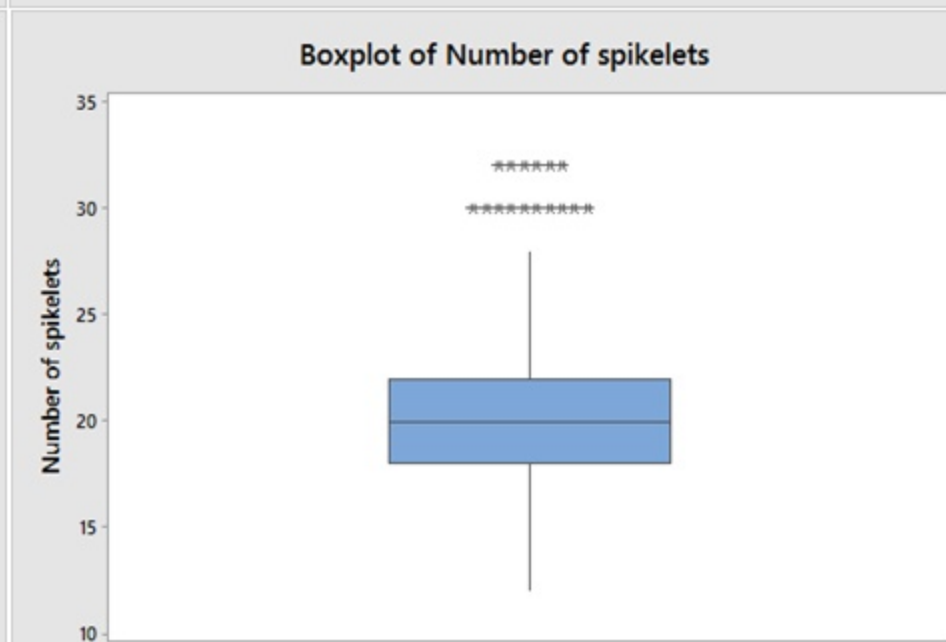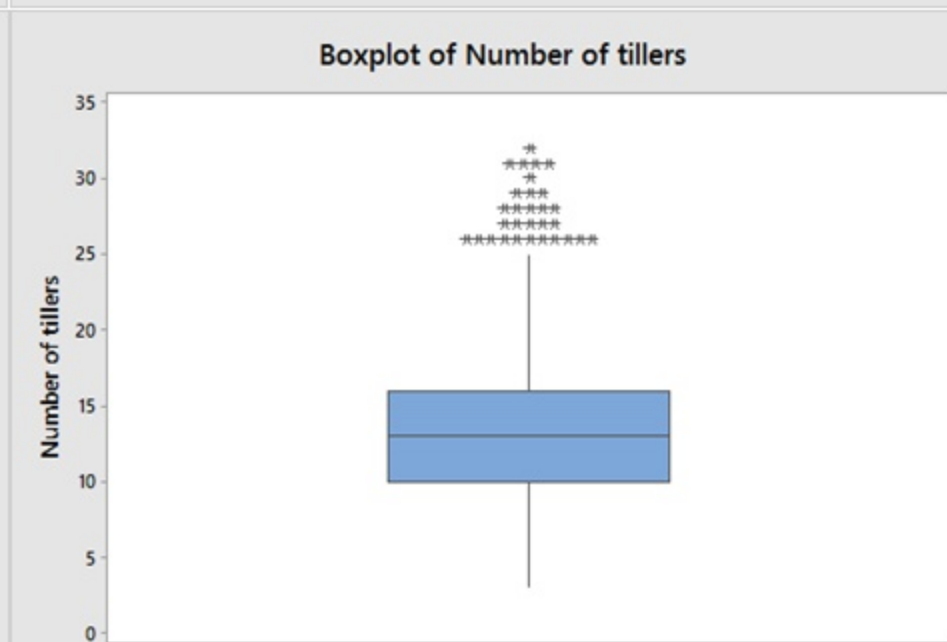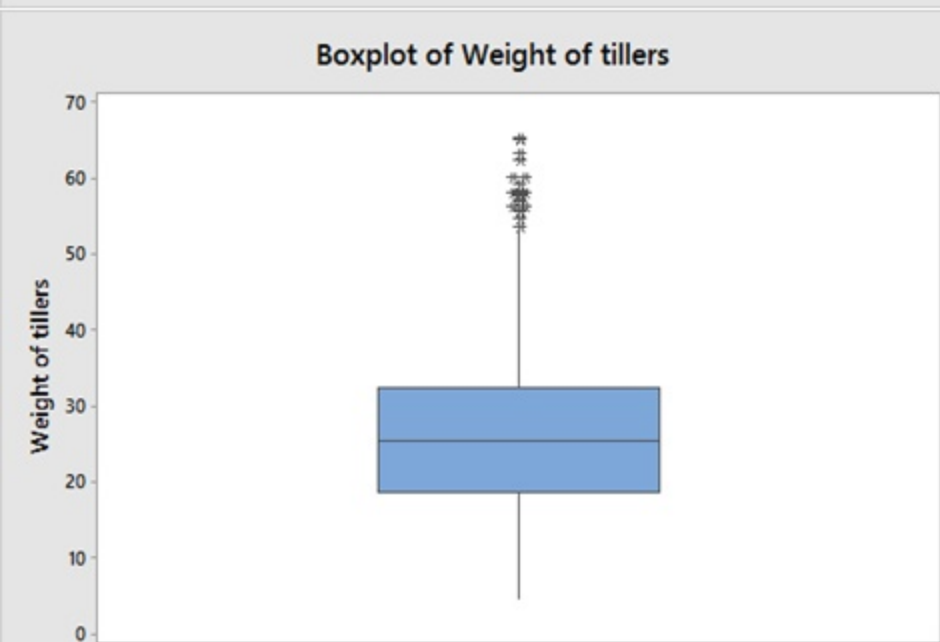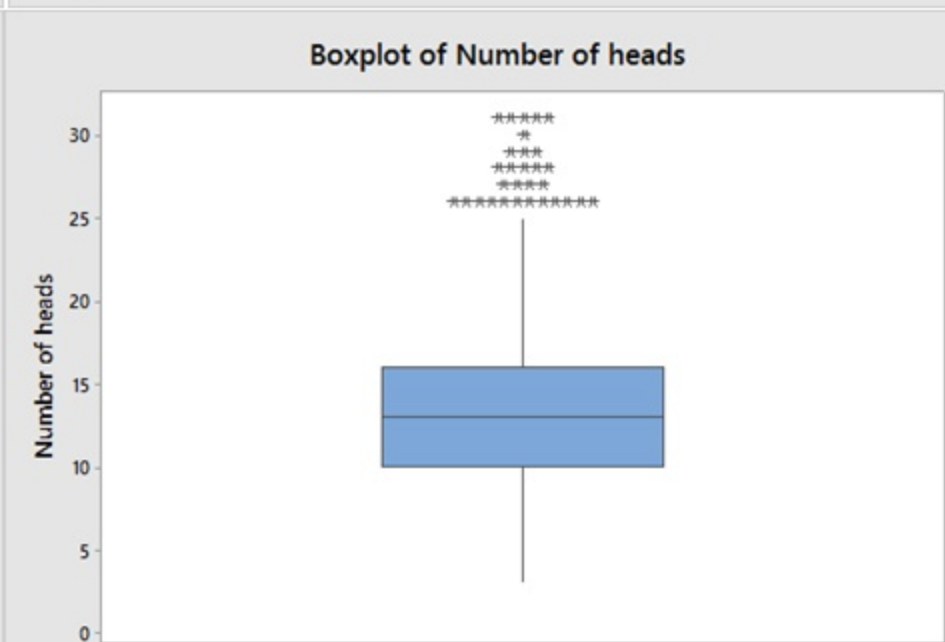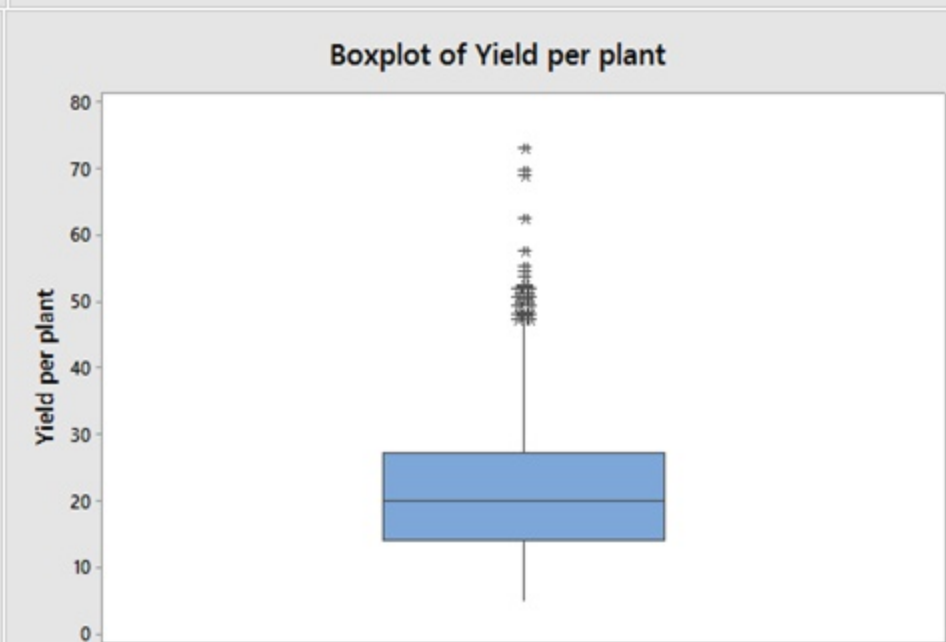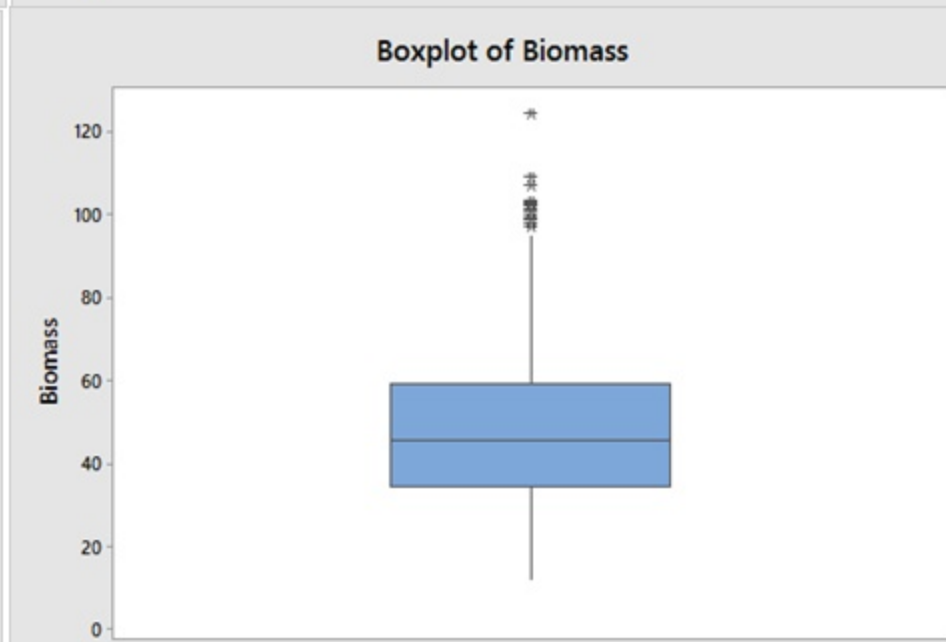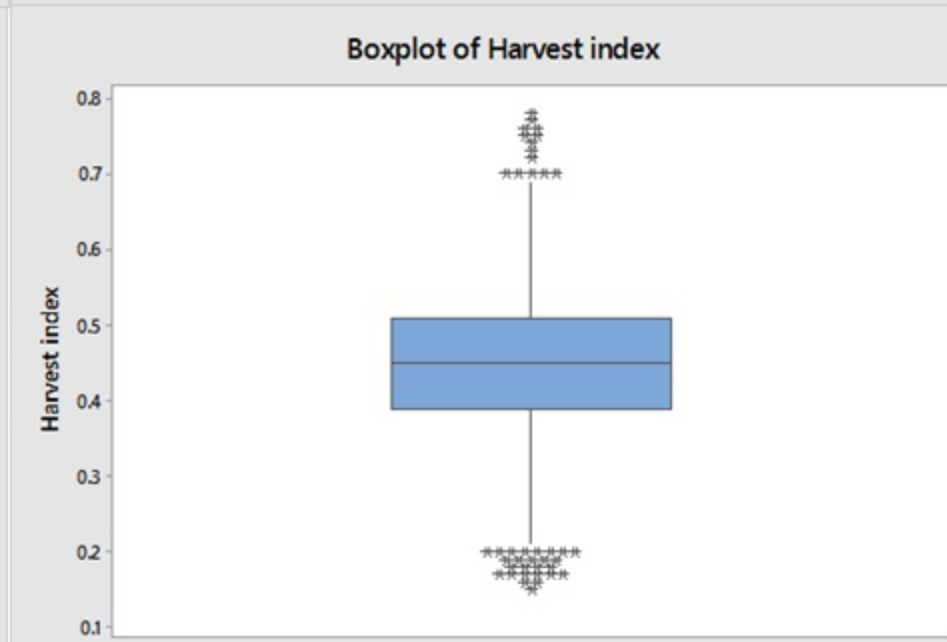

Supplement: Supplementary file 7 [file Data_Sheet_1.PDF]
