# Supplementary material for: Agro-Morphological, Yield, and Genotyping-by-Sequencing Data of Selected Wheat (Triticum aestivum) Germplasm From Pakistan
Source: Front Genet. 2021 Apr 13;12:617772. doi: 10.3389/fgene.2021.617772 (PMC8216712; doi:10.3389/fgene.2021.617772)
Supplement: Supplementary file 8 [file Data_Sheet_2.docx]

**Supplementary information**

**Quantitative data recording:**

1. Plant height/peduncle length (PH): Roots were cut at 2 inches from the soil. Plant height data shows peduncle length (cm) of the longest tiller from its root to the base of the spike.
2. Number of nodes per tiller (NN): Numbers of nodes were counted on the longest tiller of all individual plants.
3. Number of spikelets per spike (NS): For the spike on the longest tiller, total spikelets were counted for all genotypes.
4. Number of tillers per plant (NT): Before cutting the roots, numbers of tillers per plant originating from the same root were counted.
5. Weight of tillers (WT): After cutting the roots and spikes from all tillers, weight (in grams) was recorded. This variable represents total weight per plant excluding the weight of heads/spikes.
6. Number of heads/spikes per plant (NH): Numbers of spikes or heads were counted for all genotypes. In most cases, this number corresponded to the total number of tillers and is a measure of the number of reproductive tillers.
7. Yield per plant (YP): Seeds collectively contained in all spikes of an individual plant were threshed separately. The total weight (in grams) of the grains produced by tall spikes of one plant was recorded.
8. Biomass (B): Biomass (in grams) was calculated as the sum of the weight of tillers (WT) and yield per plant (YP).
9. Harvest Index (HI): Harvest index was calculated as the ratio of yield per plant (YP) to biomass (B), as reported by Dai et al (Dai et al., 2016).

Technical validation:

Seven cultivars were included as duplicate controls in the current study for technical validation. These include Sahar (Genotype IDs: 37 and 143), Faisalabad 2008 (Genotype IDs: 38 and 140), Lasani 2008 (Genotype IDs: 39 and 144), Marvi 2000 (Genotype IDs: 46 and 147), Chakwal 50 (Genotype IDs: 49 and 114), Galaxy (Genotype IDs: 54 and 141), and TD-1 (Genotype IDs: 52, 131). One replicate for these genotypes (genotype IDs: 37, 38, 39, 46, 49, 52, and 54) was collected from Cereal Crops Research Institute (CCRI), Pirsabak, Nowshera, while the second replicate was collected from different research institutes: genotype 114 from Barani Agricultural Research Institute (BARI), Chakwal; genotypes 140, 141, 143, and 144 from Federal Seed Certification and Registration Department (FSC&RD), Khanewal and genotypes 131 and 147 from Nuclear Institute of Agriculture (NIA), Tandojam. These duplicated genotypes were randomly assigned separate genotype IDs and sown in the field like other genotypes. Their agro-morphological and yield data were subjected to multivariate analyses including principal component analysis (PCA) and hierarchical cluster analysis or dendrogram in Minitab version 18. Fig. 1C is the PCA plot using average values of 15 samples per genotype, while Fig. 1D shows the dendrogram of these seven genotypes. These figures show that, except for Chakwal 50 (Genotype IDs: 49 and 114), all duplicated genotypes tend to cluster together, but appear distinct from other cultivars. This observation attests to the authenticity of the agro-morphological data. Chakwal 50 replicates (Genotype IDs: 49 and 114) appeared very distinct from each other (Fig. 1C and Fig. 1D). They tend to cluster with other genotypes rather than clustering together. Due to discordance in morphological results among the replicates, both replicates (49 and 114) were subsequently selected to generate GBS data.

Using the SNPs generated from the GBS data (provided in Genotyping and SNPs data file (Islam et al., 2020), values of Pearson Correlations between the two alleles within each replicate and between the two replicates were calculated using Minitab version 18 (Table 1). Almost perfect correlations between alleles 1 and 2 in each genotype (above 0.99 correlation values) reflected high genomic homozygosity within each replicate, nullifying the chances of mixing distinct genotypes in original DNA extractions intended for GBS. On the other hand, moderate correlations (less than 0.5) between the two replicates revealed distinctness between them. Together with the agro-morphological findings, the two replicates used for Chakwal-50 were two distinct genotypes rather than the two replicates of a single genotype. The exact identification of these two genotypes (49 and 114) could not be established from current data.

For generating GBS data, DNA from the selected genotypes was extracted after mixing young fresh growing leaves of 10 seedlings per genotype, to ensure that the sequencing data was representative of the genotype and not any individual plant. High quality of the sequencing data was evident in FastQC analyses; up to 90% of all the short reads exhibited a Phred quality score of Q30 (99.9% correct base calling) or above. SNPs were called for variants having a minimum of tag4 value (coverage depth of 4 or more) and a quality score of Q20 (99% accuracy) or more. Thus, only high-quality variants were included in the dataset. The authenticity of GBS data was evaluated by selecting 16,000 SNPs from the Genotyping and SNPs data file (Islam et al., 2020). These SNPs were selected using the following criteria: (a) presence of alleles in all genotypes (zero missing data), (b) common alleles among the genotypes (more than 0.3 minor allele frequency), and (c) representation of SNPs from all 42 chromosomes in the wheat genome. From these SNPs, a dendrogram was generated in the R program to show the relationship among the genotypes. Two distinct clusters were evident in the dendrogram whereby genotypes belonging to different sources of collections tended to cluster together (Fig. 1E). This approach not only validated the usefulness of GBS data but also made obvious the genetic distinctness of the genotypes collected by the institutes (original sources of sample collection for this study).
